# The Multiplicity and Confusion of Infections

**Published:** 1918-06

**Authors:** 


**A Monthly Review of Medicine and Surgery**

EDITOR AND PUBLISHER, DR. A. L. BENEDICT, 228 Summer St., corner of Elmwood Ave., Buffalo, (Address for all communications. Please make personal and telephone calls before 1 P. M.)

Third, in age, on the western continent. Independent, but supports professional organizations. News limited mainly to Western N. Y. and Penn.

Subscription $2.00 a year; $3.00 for two years in advance. Changes and errors in address or failure or duplication of delivery, should be reported immediately.

Yearly Volume 73 JUNE, 1918 Number 11

### The Multiplicity and Confusion of Infections.

As has appeared from numerous abstracts, a number of coccidial, hyphomycetic and protozoic micro-organisms have either been discovered or recognized as pathogenic within the last decade or so. The pneumococcus has been shown to have three distinct types, not to mention the fourth group which is a sort of scrap basket for various bacteria, and the habit of bacteriologists of reporting "atypic types." Human tuberculosis has been separated into two quite distinct infections, with relatively permanent varieties, of human and bovine types. Dysentery has not only been separated into bacillar and amoebic types but at least two distinct strains of the former have been described, while it is not altogether certain that the latter is entirely due to a single kind of amoeba and even amoebae described as non-pathogenic are somewhat under suspicion. Clinical typhoid has been separated into the true infection and two comparatively common para-infections, which shade into pathogenic processes due to the colon bacillus. The gas bacillus has grown from a newly described bacterium into an organism of practical importance, recognizable to a large degree by symptomatology. Various infections, long recognized as specific, but with no knowledge as to their germ, have been claimed to be due to certain newly identified ones, some with practical certainty, as syphilis, others with more or less dispute as to the validity of the bacteriologic claims. Yellow fever has been definitely cleared up so far as the means of conveyance and identity of the carrier are concerned but the real germ remains unknown, though various claims of its discovery have been made. Malaria has been re-classified, with a general corroboration of the validity of the old clinical classification but quotidian fever explained as double tertian infection. In fact, except for certain clinical and biologic analogies, malaria can no longer be considered as one infection but rather as several, quite as distinct as typhus and typhoid which were quite recently confused and as the venereal diseases which the 17th century physicians confused in a manner almost incomprehensible when we consider their usual accuracy in regard to gross clinical manifestations. A considerable number of animal invading organisms have been discovered quite recently, from those of small size more or less accurately answering to the ordinary definition of infections, up to macroscopic parasites whose lesions, after all, allow no sharp line to be drawn between infections and infestations.

It is a rather remarkable fact that a considerable number of well and long known diseases, of high frequency, such as the exanthemata, remain among the list of diseases of unknown cause while various infections and infestations little known in this country and Europe, and often of rather low incidence anywhere in the world, have been definitely connected with living causes.

In several instances, apparently well demonstrated germs have been discarded as the true causes of their respective diseases—those of malignant tumors, epilepsy, mumps, rheumatism, scarlet fever, etc. Perhaps the most conspicuous instance of a discarded germ is the protozoon of variola-vaccinia, whose complicated extra and intra-corporeal genealogy was figured out in detail.

One of the problems which is being seriously attacked as a part of military medicine, is the matter of sepsis, long spoken of as non-specific. We have already reached the point at which it is possible to recognize by clinical appearance, with reasonable probability, the effects of certain pyogenic organisms and, especially since the outbreak of the war and in spite of the marked tendency in certain quarters to limit all antiseptic measures to Dakin's solution or dichloramine T, the attempt is being made to reduce to practical therapeutics, the familiar bacteriologic tables that this or that antiseptic reduced one bacterium at one rate and another at another. It is not entirely a problem of research along new paths but to a considerable degree one of analyzing and making practical deductions from information already available, to separate the various pyogenic infections, to recognize their clinical features and different severities, and to select for each, the optimum method of treatment.

All this is confusing at present. We are confronted with the necessity of differential diagnosis among a far greater number of living causes of disease than formerly. The problem is not merely one of academic interest, but of practical importance. In a sense, it is merely in regard to prognosis that the practical issue arises in many infections formerly confused. But, as for the distinction between typhoid and the paratyphoids and the types of pneumococcus, the responsibility and even the therapeutic guidance of the physician is decidedly different, if we contrast the old conception that he had say 100 cases of a given disease out of which he was, so to speak, allowed to lose 10, with the new conception that he has—without attempting accuracy of numbers—70 cases of one disease, 20 of another and 10 of a third, quite distinct though presenting clinical resemblances, and that he might be satisfied with losing 10 of the first, 1 of the second and none of the third. Even though such factors as confinement to bed, diet and medication are nearly the same for all three, in general terms, the intensity of medical and nursing care appropriate for diseases of different severity is decidedly different. Moreover, the physician who is attempting to increase his efficiency, will feel that even the general acceptance of generally identical methods for three or four diseases is only a temporary matter, especially when we consider the very different methods of therapeutics already established for various diseases more or less anciently confused, and the discriminations already beginning to be made in regard to the septic infections and various other distinctions of types of disease still known by a single name. Thus, even if the bacteriologic differentiation involved merely differences in prognosis, it would still be important, for day-after-to-morrow if not for today but the differentiation already involves more important points than prognosis in some instances. This general statement means detailed problems of considerable difficulty, in regard to the interrelations of the profession, and of the relations of the laity to the different professional men involved, even economic problems, simple enough for the very rich and the very poor, but requiring serious study for the great mass of the honest and independent but by no means wealthy population.

The increased confusion of nomenclature and conception of disease is largely due to the discovery of new germs and the separation of diseases formerly considered units into types which may really be subdivisions of a unit group corresponding to varieties of a species of germs, or which may be theoretically and practically separate diseases, corresponding to entirely distinct germs which merely happen to present analogies in their methods of parasitism. This confusion may be greatly simplified if we cut loose from all such terms as pneumonia, meningitis and the like, or else use each term in a purely locational and histologic sense or else as indicative of an infection by a given germ, without regard to where or how it invades the body. Indeed, with special reference to carriers, it may be well to ignore the question of whether it causes disease at all or merely lodges somewhere in or on the body, though, as discussed previously, there are radical differences of kind among carriers, the extremes being represented by the old fashioned physician carrying the cause of measles on his whiskers from house to house, and by Typhoid Mary. A most illuminating suggestion, partially backed by facts, has recently been made as to the rarity of menigitis meaning meningococcus meningitis, or of anterior poliomyelitis of at least putative specific nature, in epidemics. This suggestion is to the effect that there are not merely typic cases and carriers, that the disease is not merely conveyed, once in a while, by some particular route of infection such as the nares and the communicating sinuses, but that the disease actually does occur in numerous cases during an epidemic, like more familiar infections such as the exanthemata, but that it usually occurs in mild type, perhaps as an influenza—again a term of very different meanings—or a mere coryza, with occasional involvements now alone recognized, of high mortality. Such a conception is quite in keeping with the fact, itself not so well comprehended as it should be, that pneumococcic infection quite often assumes the meningeal type, with the well recognized fact that tuberculosis presents tremendous differences according to its location and the resistance of the individual, with the fact that even such a disease as gonococcus infection which is usually true to its clinical type, may occur as a septicaemia or involvement of the meninges.

To clear up the confusion, we must coin new terms or give definite meanings to old ones, to indicate 1. the gross anatomic location of an infection; 2. its histologic and functional perversions; 3. the identity of the organism. All these points must be covered in the diagnosis.

---

**Sodoku.** Felix Ramond and Lévi-Bruhl, report a case, Le Prog. Med., Dec. 29, 1917, and state that an observer not seeing the case till the second phase had developed, would diagnose it as trench fever. In view of the common occurrence of rat bite in the trenches, they raise the question whether the latter condition is not due to the conveyance of the suppositious specific micro-organism by this means.

